# Biophysics and systems biology

**DOI:** 10.1098/rsta.2009.0245

**Published:** 2010-03-13

**Authors:** Denis Noble

**Affiliations:** Department of Physiology, Anatomy and Genetics, University of Oxford, Parks Road, Oxford OX1 3PT, UK

**Keywords:** cell biophysics, systems biology, computational biology, mathematical biology

## Abstract

Biophysics at the systems level, as distinct from molecular biophysics, acquired its most famous paradigm in the work of Hodgkin and Huxley, who integrated their equations for the nerve impulse in 1952. Their approach has since been extended to other organs of the body, notably including the heart. The modern field of computational biology has expanded rapidly during the first decade of the twenty-first century and, through its contribution to what is now called systems biology, it is set to revise many of the fundamental principles of biology, including the relations between genotypes and phenotypes. Evolutionary theory, in particular, will require re-assessment. To succeed in this, computational and systems biology will need to develop the theoretical framework required to deal with multilevel interactions. While computational power is necessary, and is forthcoming, it is not sufficient. We will also require mathematical insight, perhaps of a nature we have not yet identified. This article is therefore also a challenge to mathematicians to develop such insights.

## 1. Introduction: the origins of biophysics and systems biology

As a young PhD student at University College London, I witnessed the celebrations of the 300th anniversary of the Royal Society in 1960. As the magnificent procession of red-gowned Fellows of the Royal Society (FRS) paraded into the Royal Albert Hall, two black gowns suddenly appeared. They were worn by Alan Hodgkin and Andrew Huxley. The founders of the field of cellular biophysics, with their ground-breaking mathematical reconstruction of the nerve impulse (Hodgkin & Huxley 1952), were simply Mr Hodgkin and Mr Huxley—neither had submitted a thesis for a PhD. With ‘FRS’ to their names, they hardly needed to! A year later, Alan Hodgkin examined my PhD thesis, which applied their ideas to reconstructing the electrical functioning of the heart (Noble 1960, 1962), and 3 years later we were celebrating their Nobel Prize.

It is highly appropriate to recall these events in a volume to celebrate the 350th anniversary, but they also remind us that the field that is now called systems biology has important historical roots. Hodgkin and Huxley themselves were not the first. I would nominate Claude Bernard as the first systems biologist (Noble 2008*a*), since in the middle of the nineteenth century he formulated the systems principle of control of the internal environment (Bernard 1865). This is well known and is widely recognized as the homeostatic basis of modern physiological science. It is much less well known that Bernard also presaged the development of mathematical biology when he wrote ‘this application of mathematics to natural phenomena is the aim of all science, because the expression of the laws of phenomena should always be mathematical.’^1^ Other historical roots can be found in the work of Harvey (Auffray & Noble 2009) and Mendel (Auffray 2005). Despite these strong historical roots, however, the field did not flourish in the second half of the twentieth century. Soon after Hodgkin and Huxley’s achievement it was to be swept aside as molecular biology took the centre stage.

## 2. The achievements and problems of molecular biology

Physicists and mathematicians contributed greatly to the spectacular growth of molecular biology. The double-helical structure of DNA was discovered in the Cavendish laboratory in Cambridge (Watson & Crick 1953*a*,*b*) and in the biophysics laboratory at King’s College London (Franklin & Gosling 1953*a*,*b*; Wilkins *et al*. 1953), while some of the seminal ideas of molecular biology were first developed by Schrödinger (1944). In addition to correctly predicting that the genetic material would be found to be an aperiodic crystal, his book, *What is Life?,* followed a proposal by Max Delbrück (see Dronamrajua 1999) that was to prove fundamental in the twentieth century interpretation of molecular biology. This was that physics and biology are essentially different disciplines in that while physics is about the emergence of order from disorder, such as the ordered global behaviour of a gas from the disordered Brownian motion of the individual molecules, biology dealt with order even at the molecular level. The paradigm for this view was the effects of mutations of the genetic material. Even a single switch from one nucleotide to another, corresponding to a single amino acid change in the protein for which the DNA sequence acts as a template, can have dramatic effects on the phenotype at higher levels. A good example in the case of the heart is that of the various sodium channel mutations that can cause arrhythmia (Clancy & Rudy 1999), and there are excellent examples in the processes of embryonic development (Davidson 2006).

The attribution of control to the DNA was strongly reinforced by Monod and Jacob (Jacob *et al*. 1960), who interpreted their work as evidence for the existence of a ‘genetic program’, an analogy explicitly based on comparison with an electronic computer: ‘The programme is a model borrowed from electronic computers. It equates the genetic material with the magnetic tape of a computer’ (Jacob 1982), while the rest of the organism, particularly the fertilized egg cell, could be compared with the computer itself. Specific instructions at the level of DNA could then be seen to ‘program’ or control the development and behaviour of the organism. These ideas married well with the gene-centred theories of evolution and the metaphor of ‘selfish’ genes (Dawkins 1976, 1982, 2006), which relegated the organism to the role of a disposable transient carrier of its DNA.

It is not surprising therefore that the peak of the achievement of molecular biology, the sequencing of the complete human genome, was widely signalled as finally reading the ‘book of life’. However, the main architects of that project are much more circumspect: ‘One of the most profound discoveries I have made in all my research is that you cannot define a human life or any life based on DNA alone…’. Why? Because ‘An organism’s environment is ultimately as unique as its genetic code’ (Venter 2007). Sulston is also cautious: ‘The complexity of control, overlaid by the unique experience of each individual, means that we must continue to treat every human as unique and special, and not imagine that we can predict the course of a human life other than in broad terms’ (Sulston & Ferry 2002). So also is Sydney Brenner, whose work has contributed so much to the field: ‘I believe very strongly that the fundamental unit, the correct level of abstraction, is the cell and not the genome’ (lecture at Columbia University 2003).

I have briefly summarized some of these aspects of the development of molecular biology because, in fulfilling my brief to look into the crystal ball and give my own perspective on where my subject is heading in the next 50 years, I am going to turn some of the concepts derived from the successes of molecular biology upside down. I suggest that the next stage in the development of biological science will be revolutionary in its conceptual foundations (Shapiro 2005; see also Saks *et al*. 2009) and strongly mathematical in its methods. I also see this as the fulfilment of Claude Bernard’s dream of the role of mathematics in his discipline, a dream that certainly could not be achieved in his lifetime.

## 3. Digital, analogue and stochastic genetic causes

Since the C, G, A, T sequences can be represented digitally (two bits are sufficient to represent four different entities, so the three billion base pairs could be represented by six billion bits), the idea of a determinate genetic program in the DNA, controlling the development and functioning of the organism, rather like the digital code of a computer program, was seductive, but for it to be correct, three conditions need to be satisfied. The first is that the relevant program logic should actually be found in the DNA sequences. The second is that this should control the production of proteins. The third is that this should be a determinate process. It is now known that none of these conditions are fulfilled. Molecular biology itself has revealed these deficiencies in at least six different ways.

(i) The C, G, A, T sequences of nucleotides in the genome do not themselves form a program as normally understood, with complete logic (i.e. one that could be subjected to syntactic analysis) of a kind that could separately run a computer. We cannot therefore predict life using these sequences alone. Instead, the sequences form a large set of templates that the cell uses to make specific proteins, and a smaller bank of switches, the regulatory genes, forming about 10 per cent of human genes, and the regulatory sites on which the regulatory proteins and other molecules act. Impressive switching circuits can be drawn to represent these (Levine & Davidson 2005). But they require much more than the DNA sequences themselves to operate since those switches depend on input from the rest of the organism, and from the environment. Organisms are interaction machines, not Turing machines (Shapiro 2005; Neuman 2008; Noble 2008*c*). There is therefore no computer into which we could insert the DNA sequences to generate life, other than life itself. Far from being just a transient vehicle, the organism itself contains the key to interpreting its DNA, and so to give it meaning. I will return later to this question (see §7).
(ii) In higher organisms, the sequences are broken into sometimes widely dispersed fragments, the exons, which can be combined in different ways to form templates for many different proteins. Something else must then determine which combination is used, which protein is formed and at which time. The DNA sequences therefore better resemble a database on which the system draws rather than a logical program of instructions (Atlan & Koppel 1990; Shapiro 2005; Noble 2006). For that we must look elsewhere, if indeed it exists at all. The dispersed nature of the exons and the combinatorial way in which they are used also challenges the concept of genes as discrete DNA sequences (Keller 2000*a*; Pearson 2006; Scherrer & Jost 2007).
(iii) What determines which proteins are made and in what quantity is not the DNA alone. Different cells and tissues use precisely the same DNA to produce widely different patterns of gene expression. This is what makes a heart cell different from, say, a bone cell or a pancreatic cell. These instructions come from the cells and tissues themselves, in the form of varying levels of transcription factors and epigenetic marks (Bird 2007) that are specific to the different types of cell. These processes are robust and inherited. Differentiated heart cells always form new heart cells as the heart develops, not new bone cells. They would need to be ‘de-differentiated’ to form multipotent stem cells in order to give rise to a different differentiated cell. This should not surprise us. Some kinds of cellular inheritance, perhaps starting with the ability of a lipid membrane-enclosed globule to divide, almost certainly predated genome inheritance (Maynard Smith & Szathmáry 1995).
(iv) The resulting patterns of gene expression are not only widely variable from one tissue to another, they themselves are not digital. The expression levels vary continuously in a way that is better described as an analogue. Since we must include these analogue levels in any description of how the process works, any ‘program’ we might identify is not based on digital coding alone. It is significant therefore that the inclusion of analogue processing is seen by some computer scientists as an important way in which a system can perform beyond the Turing limits (Siegelmann 1995, 1998, 1999). Organisms are, at the least, ‘super-Turing’ machines in this sense.
(v) Gene expression is a stochastic process (Kaern *et al*. 2005). Even within the same tissue, there are large variations in gene expression levels in different cells. Such stochasticity is incompatible with the operation of a determinate Turing machine (Kupiec 2008; Neuman 2008).
(vi) Finally, there is continuous interaction between DNA and its environment. As Barbara McClintock put it in her Nobel prize lecture (1983) for her work on ‘jumping genes’, the genome is better viewed as ‘a highly sensitive organ of the cell’ that can be reorganized in response to challenges (Keller 1983). We now also understand the extent to which organisms can swap DNA between each other, particularly in the world of micro-organisms (Goldenfeld & Woese 2007).

Another way to express the significance of these developments in molecular biology is to say that not much is left of the so-called ‘central dogma of biology’ (see Shapiro (2009) for more details) other than that part of Crick’s original statement of it that is correct, which is that while DNA is a template for amino acid sequences in proteins, proteins do not form a template from which DNA can be produced by a reverse version of the DNA→protein transcription process. But in the extended sense in which it is frequently used in a neo-Darwinist context, as forbidding the passage of information from the organism and environment to DNA, the ‘dogma’ is seriously incorrect. Information is continually flowing in the opposite direction. I will return later to the significance of this fact for neo-Darwinism itself.

To these facts we must add a few more before we reassess the comparison between physics and biology.

(vii) Many genetic changes, either knockouts or mutations, appear not to have significant phenotypic effects; or rather they have effects that are subtle, often revealed only when the organism is under stress. For example, complete deletion of genes in yeast has no obvious phenotypic effect in 80 per cent of cases. Yet, 97 per cent have an effect on growth during stress (Hillenmeyer *et al*. 2008). The reason is that changes at the level of the genome are frequently buffered, i.e. alternative processes kick in at lower levels (such as gene–protein networks) to ensure continued functionality at higher levels (such as cells, tissues and organs). And even when a phenotype change does occur there is no guarantee that its magnitude reveals the full quantitative contribution of that particular gene since the magnitude of the effect may also be buffered. This is a problem I have recently referred to as the ‘genetic differential effect problem’ (Noble 2008*c*) and it has of course been known for many years. There is nothing new about the existence of the problem. What is new is that gene knockouts have revealed how extensive the problem is. Moreover, there is a possible solution to the problem to which I will return later.
(viii) The existence of stochastic gene expression allows some form of selection operating at the level of tissues and organs (Laforge *et al*. 2004; Kaern *et al*. 2005; Kupiec 2008, 2009). In fact, such selection may be a prerequisite of successful living systems which can use only those variations that are fit for purpose. As Kupiec has noted, Darwinian selection could also be very effective within the individual organism, as well as between organisms.
(ix) Not only is gene expression stochastic, the products of gene expression, the proteins, each have many interactions (at least dozens) with other elements in the organism. Proteins are not as highly specific as was once anticipated. Bray (Bray & Lay 1994; Bray 2009) has highlighted the role of multiple interactions in comparing the evolution of protein networks with that of neural networks.

## 4. The multifactorial nature of biological functions

So, while it is true to say that changes at the molecular level can *sometimes* have large effects at the higher phenotype levels, these effects are frequently buffered. Even the sodium channel mutations I referred to earlier do not, by themselves, trigger cardiac arrhythmia. The picture that emerges is that of a multifactorial system. Biology, it turns out, must also create order from stochastic processes at the lower level (Auffray *et al*. 2003). Physics and biology do not after all differ in quite the way that Schrödinger thought. This is a point that has been forcibly argued recently by Kupiec (2008, 2009). There is absolutely no way in which biological systems could be immune from the stochasticity that is inherent in Brownian motion itself. It is essential therefore that biological theory, like physical theory, should take this into account.

The systems approach has already pointed the way to achieve this. The massively combinatorial nature of biological interactions could have evolved precisely to overcome stochastic effects at the molecular level (Shapiro 2009). As Bray (2009) notes, protein networks have many features in common with the neural networks developed by artificial intelligence researchers. They can ‘evolve’ effective behaviour strategies from networks initialized with purely random connections, and once they have ‘evolved’ they show a high degree of tolerance when individual components are ‘knocked out’. There is then what Bray calls ‘graceful degradation’, which can take various forms (not necessarily requiring random connectivity). This provides an insight into the nature of the robustness of biological systems. Far from stochasticity being a problem, it is actually an advantage as the system evolves. ‘Graceful degradation’ is also a good description of what happens in knockout organisms. All may appear to be well when the organism is well-fed and protected. The deficiency may reveal itself only when the conditions are hostile.

I suspect that more relevant insights will come from analysis of such artificial networks and even more so from the modelling of real biological networks. Note that such networks do not require a separate ‘program’ to operate. The learning process in the case of artificial networks, and evolutionary interaction with the environment in the case of biological networks, is the ‘programming’ of the system. So, if we still wish to use the program metaphor, it is important to recognize that the program is the system itself (Noble 2008*c*). The plant geneticist Enrico Coen expressed this point well when he wrote ‘Organisms are not simply manufactured according to a set of instructions. There is no easy way to separate instructions from the process of carrying them out, to distinguish plan from execution’ (Coen 1999). This is another version of the points made earlier about the limitations of regarding the DNA sequences as a program.

## 5. The multilevel nature of biological functions

This takes me to the question of multilevel analysis. Organisms are not simply protein soups. Biological functions are integrated at many different levels. Thus, pacemaker rhythm in the heart is integrated at the level of the cell. There is no oscillator at the biochemical level of subcellular protein networks (Noble 2006). Tempting though it may be to think so, there is therefore no ‘gene for’ pacemaker rhythm. A set of genes, or more correctly the proteins formed from their templates, is involved, together with the cellular architecture—and which set we choose to represent depends on the nature of the questions we are asking. But that does not prevent us from building computer programs that mimic pacemaker rhythm. Simulation of cardiac activity has been developed over a period of nearly five decades and is now sufficiently highly developed that it can be used in the pharmaceutical industry to clarify the actions of drugs (Noble 2008*b*).

Does not the fact that we can succeed in doing this prove that, after all, there are genetic programs? Well no, for two reasons. First the logic represented by such computer simulation programs is certainly not to be found simply in the DNA sequences. The programs are representations of the processes involved at *all* the relevant biological levels, right up to and including the intricate architecture of the cell itself. And when even higher levels are modelled, the structural biology included is that of tissues or the entire organ (Hunter *et al*. 2003; Garny *et al*. 2005). In the case of the heart, the three-dimensional imaging technology to achieve this has now advanced to paracellular or even subcellular levels (Plank *et al*. 2009).

Second, reflecting Coen’s point above, the processes represented in our modelling programs *are* the functionality itself. To the extent that the program succeeds in reproducing the behaviour of the biological system it reveals the *processes* involved, not a separate set of *instructions*.

Multilevel simulation will be a major development in biology as the project known as the Human Physiome Project develops. Recent issues of this journal have been devoted to one of its components, the Virtual Physiological Human (VPH) project (Clapworthy *et al*. 2008; Fenner *et al*. 2008) and some of the achievements and future challenges of the Physiome Project (Bassingthwaighte *et al*. 2009) and its relation to systems biology (Kohl & Noble 2009) have recently been reviewed.

## 6. A theory of biological relativity?

One of the major theoretical outcomes of multilevel modelling is that causation in biological systems runs in both directions: upwards from the genome and downwards from all other levels.^2^ There are feedforward and feedback loops between the different levels. Developing the mathematical and computational tools to deal with these multiple causation loops is itself a major challenge. The mathematics that naturally suits one level may be very different from that for another level. Connecting levels is not therefore trivial. Nor are the problems simply mathematical and computational. They also require biological insight to determine how much detail at one level is relevant to functionality at other levels. These problems are now exercising the minds of interdisciplinary teams of researchers involved in the Physiome Project and they offer great opportunities for physical and mathematical scientists in the future. They have also led some physicists and biologists to develop what might be called theories of biological relativity. My own version of this idea is that, in multilevel systems, there is no privileged level of causation (Noble 2008*a*,*c*). Others have also pointed out that such a principle need not be restricted to biological systems. It could become a general theory of relativity of levels. Such a theory, called scale relativity (Nottale 1993, 2000), already exists in physics and its possible applications to biological systems have been the subject of major recent reviews (Auffray & Nottale 2008; Nottale & Auffray 2008).

I will not review these theories in detail here. I wish rather to draw attention to a related general question. Is multilevel analysis simply a matter of including downward causation (Noble 2006)? And what exactly do we mean by that term?

In my own field the paradigm example originated with Alan Hodgkin. The proteins that form ion channels in excitable cells generate electric current that charges or discharges the cell capacitance. That can be seen as upward causation. But the electrical potential of the cell also controls the gating of the ion channel proteins. This downward causation closes the loop of the ‘Hodgkin cycle’.

Is downward causation always discrete feedback or feedforward? The answer is no and the basis for that answer is profound, forming one of the reasons why I think that systems biology is revolutionary. A feedback loop can be closed. Feedback loops could exist between the levels of an organism, while the organism itself could still be modelled as a closed system. Yet, we know that organisms are not closed systems. Firstly they exchange energy and matter with the environment, including particularly other organisms whose existence forms a major part of the selection pressure. That is well recognized as a reason for regarding organisms as open systems. But there are other reasons also. I think that the best way to explain that is mathematical.

We model many biological processes as systems of differential equations. These equations describe the rates at which those processes occur. The number of such equations depends on the kind of question we are asking. At a cellular or subcellular (protein network) level, there may be a few dozen equations for the protein and other chemical entities involved. When we include structural details at the tissue or organ level, we may be dealing with millions of equations. Whatever the number, there is an inescapable requirement before we can begin to solve the equations. We must know or make plausible guesses for the initial and boundary conditions. They are not set by the differential equations themselves. These conditions restrain the solutions that are possible. In fact, beyond a certain level of complexity, the more interesting question becomes the explanation of that restraining set of conditions, not just the behaviour of the system, since the restraints may completely change the behaviour of the system. A restraint, therefore, is not necessarily a feedback. Restraints can be simply the background set of conditions within which the system operates, i.e. its environment. Through these interactions organisms can adapt to many different conditions. Their robustness in doing so distinguishes them from complex nonlinear systems that are highly sensitive to initial conditions or which end up unable to escape attractors.

## 7. ‘Genetic programs’

This is a suitable point at which to return to the question of ‘genetic programs’. As we have seen, DNA sequences act as templates for proteins and as switches for turning genes on and off when they are in an organism, starting with the fertilized egg cell and maternal environment in the case of higher animals. A possible objection to my conclusion that the DNA sequences are better viewed as a database rather than as a program is that all programs require a computer to implement them. It was part of Monod and Jacob’s idea that, if DNA is the program, the organism is equivalent to the computer. Programs also do nothing outside the context of a computer. Could we somehow update this approach to save the ‘program’ metaphor? It is so ingrained into modern thought, among laypeople as well as most scientists, that it may now be difficult to convince people to abandon it. It is therefore worth spelling out, once again, what the difficulties are.

DNA sequences alone are not capable of being parsed as the complete logic of a program. Whenever we talk of a genetic program we must also include steps that involve the rest of the organism (e.g. my discussion of the ‘circadian rhythm’ program in Noble (2006, pp. 69–73), and this is certainly true for the analysis of cardiac rhythm (Noble 2006, pp. 56–65)). Much of the logic of living systems lies beyond DNA. To save the program metaphor therefore we would have to say that the ‘program’ is distributed between the tape and the machine. This would, incidentally, explain an important fact. Virtually all attempts at cross-species cloning fail to develop to the adult (Chung *et al*. 2009). A possible explanation is that the egg cell information is too specific (Chen *et al*. 2006). In fact, in the only case so far, that of a carp nucleus and goldfish egg, the egg cytoplasm clearly influences the phenotype (Sun *et al*. 2005). Strathmann (1993) also refers to the influence of the egg cytoplasm on gene expression during early development as one of the impediments to hybridization in an evolutionary context. There is no good reason why cells themselves should have ceased to evolve once genomes arose. But if we need a specific (special purpose) ‘computer’ for each ‘program’, the program concept loses much of its attraction.

The way to save the genetic program idea would therefore be to abandon the identification of genes with specific sequences of DNA alone and return to the original idea of genes as the causes of particular phenotypes (Kitcher 1982; Mayr 1982; Dupré 1993; Pichot 1999; Keller 2000*b*; Noble 2008*c*) by including other relevant processes in the organism. The problem with this approach is that the closer we get to characterizing the ‘program’ for a particular phenotype, the more it looks like the functionality itself. Thus, the process of cardiac rhythm can be represented as such a ‘program’ (indeed, modellers write computer programs to reproduce the process), but it is not a sequence of instructions separate from the functionality itself. This is another way to understand the quotation from Coen referred to earlier. The clear distinction between the replicator and the vehicle disappears and, with it, a fundamental aspect of the ‘selfish gene’ view.

If we do wish to retain the idea of a program, for example in talking about embryonic development where the concept of a ‘developmental program’ has its best applications (Keller 2000*a*), it might be better to think in the same terms in which we talk of neural nets being programmed. They are programmed by the initial setting up of their connections and then by the learning process, the set of restraints that allows them to ‘home in’ to a particular functionality. Those open-ended restraints are as much a part of the ‘program’ as the initial setting up of the system. The analogy with organisms as interaction machines is obvious. I am not proposing that organisms function as neural nets; only that the example of neural nets expands our concept of the word ‘program’ in a relevant way. The program is a distributed one (Siegelmann 1998) involving much more than DNA sequences, and is therefore far removed from Monod and Jacob’s original concept of a genetic program.

## 8. Systems biology and evolution

Where do the restraints come from in biological systems? Clearly, the immediate environment of the system is one source of restraint. Proteins are restrained by the cellular architecture (where they are found in or between the membrane and filament systems), cells are restrained by the tissues and organs they find themselves in (by the structure of the tissues and organs and by the intercellular signalling) and all levels are restrained by the external environment. Even these restraints though would not exhaust the list. Organisms are also a product of their evolutionary history, i.e. the interactions with past environments. These restraints are stored in two forms of inheritance—DNA and cellular. The DNA sequences restrict which amino acid sequences can be present in proteins, while the inherited cellular architecture restricts their locations, movements and reactions.

This is one of the reasons why systems biology cannot be restricted to the analysis of protein and gene circuits. The structural information is also crucial. Much of its evolution may have been independent of the cell’s own DNA since the early evolution of the eukaryotic cell involved many forms of symbiosis. The best known example is the mitochondria, which are now accepted to have originally been invading (or should we say ‘captured’?) bacteria, as were chloroplasts (Cavalier-Smith 2000, 2004). They even retain some of the original DNA, though some also migrated to the nucleus. There are other examples of symbiosis (Margulis 1981; Margulis & Sagan 2002; Williamson 2003, 2006; Williamson & Vickers 2007). Cooperativity may have been quite as important as competition in evolution (see also Goldenfeld & Woese 2007).

Cavalier-Smith has described some of these inherited features of animal and plant cells as the ‘membranome’, an important concept since lipids are not formed from DNA templates. An organism needs to inherit the membranome, which it does of course—it comes complete with the fertilized egg cell—yet another reason why it does not make sense to describe the organism as merely a vehicle for DNA. As I have argued elsewhere (Noble 2008*c*), the relative contributions of DNA and non-DNA inheritance are difficult to estimate (one is largely digital and so easy to calculate, whereas the other is analogue and hard to calculate), but the non-DNA inheritance is very substantial. It also contains many historical restraints of evolution.

This is the point at which I should attempt to explain the neo-Darwinian model and the modern synthesis and what is wrong with them from a systems viewpoint.

Neo-Darwinism brings together natural selection and nineteenth century genetics, while the modern synthesis (Huxley 1942) fuses Darwinism with twentieth century genetics. ‘Neo-Darwinism’ is the term often used for both of these syntheses. Darwin knew nothing of Mendel’s work on genetics. Moreover, he also accepted the idea of the inheritance of acquired characteristics, as did Lamarck (Lamarck 1809; Corsi 2001), who is incorrectly represented in many texts as inventing the idea. Darwin’s disagreements with Lamarck were not over the mechanisms of inheritance. Both were ignorant of those mechanisms. Their disagreement was more over the question of whether evolution had a direction or whether variation was random. Historically, we would do better to recognize Lamarck as the inventor of the term ‘biology’ as a separate science, and as championing the idea that species change (transformationism). Darwin can then be seen as discovering one of the mechanisms in his theory of natural selection, involved not only in transformations but also in the origin of species.

The problem with both revisions of Darwinism is that they involve a version of genetics that we need to revise. This version was one in which the central dogma of biology was taken to mean that the genetic material is never modified by the rest of the organism and the environment. Francis Crick’s original statements of the ‘central dogma of molecular biology’ (Crick 1958, 1970) do not in fact make such a strong claim. He stated a more limited chemical fact: that DNA sequences are used as templates to make proteins, but proteins are not used as reverse templates to make DNA. So, even if its proteins were to become modified during the lifetime of an individual, that modification cannot be inherited. The ‘dogma’ was then interpreted by many biologists to mean that information flows only one way. As we have seen, it does not. The *quantities* of proteins synthesized count as relevant information just as much as their amino acid sequences. But those quantities are most certainly dependent on signals from the rest of the system through the levels of transcription factors (including proteins and RNA) and the epigenetic marking of DNA itself and of the histone tails. All of this is open to the rest of the organism and to the environment to degrees we have yet to fully determine.

I will give just one example here to illustrate the potential significance of this openness. More examples can be found elsewhere (Jablonka & Lamb 1995, 2005). Neuroscientists have recently studied the epigenetic factors involved in maternal grooming behaviour in colonies of rats. Grooming depends on the environment. Colonies that are safe groom their young a lot. Colonies that are fighting off predators do not. This behaviour is inherited. The mechanisms are a fascinating example of epigenetic effects. The genome in the hippocampal region of the brain is epigenetically marked by the grooming behaviour and this predisposes the young to show that behaviour (Weaver *et al*. 2004, 2007). This is an important development, but as Weaver himself points out (Weaver 2009) it is currently restricted to one gene and one region of the brain. That underlines the importance of further research in this area. The implications of this form of epigenetic influence, however, are profound since it can transmit patterns of epigenetic marking through the generations even though they are not transmitted via the germline. This constitutes another form of inheritance of acquired characteristics to add to those reviewed by Jablonka and Lamb.

There is a tendency to dismiss such challenges to extensions of the central dogma as merely examples of cultural evolution. They seem to show rather that the boundaries between the different evolutionary processes are fuzzy. Once such interactions between behaviour and epigenetics are established and transmitted through the generations they can favour genetic combinations that lock them into the genome (Jablonka & Lamb 2005, pp. 260–270). This mechanism was originally described by Waddington (1942, 1957, 1959; Bard 2008), who demonstrated that, in fruitflies, just 14 generations of induced phenotype change could be assimilated into the genome. Mutations and genetic recombinations themselves are not random (Shapiro 2005). Moreover, they do not occur in a random context. They occur in the context of all the restraints exerted on the organism, including those of the environment. In such a process, it is the phenotype, not individual genes, that are the targets of selection (Keller 1999). Central building blocks of the neo-Darwinian synthesis are now known to be incompatible with the most recent discoveries in molecular biology.

## 9. Reverse engineering in systems biology

I referred earlier to the ‘genetic differential effect problem’. In a previous article in this journal I have proposed that computational systems biology could provide a solution (Noble 2008*c*). The idea is basically simple. If our understanding and simulations are good enough they should include the robustness of biological systems, including their resistance to damage from mutations and knockouts. Moreover, if the models include representations of specific gene products (i.e. they extend down to the protein level) then it should be possible to reverse engineer to arrive at *quantitative* estimates of the contribution of each gene product to the functionality represented. That may be possible even if the system completely buffers the mutation or knockout so that no effect is observed in the phenotype. I give an example of this in the previous article from work on the heart (Noble 2008*c*). However, I would readily agree that, in its present state of development, computational systems biology is a long way from being able to do this in general. But it is worth bearing this in mind as an important long-term goal.

## Footnotes

1 Cette application des mathématiques aux phénomènes naturels est le but de toute science, parce que l’expression de la loi des phénomènes doit toujours être mathématique.

2 ‘Upwards’ and ‘downwards’ in this context are metaphorical. A more neutral terminology would refer to different (larger and smaller) scales. But the concept of level is strongly entrenched in biological science so I have continued to use it here. There is also possible confusion with ‘scale’ as used in scale relativity, though I believe that one of the key questions for the future is that of relating the ideas of scale relativity to multilevel systems biology.

## References

[RSTA20090245c1] Atlan H., Koppel M. (1990). The cellular computer DNA:program or data?. Bull. Math. Biol..

[RSTA20090245c2] Auffray C. (2005). Aux sources de la biologie des systèmes et de la génétique:la pertinence des expérimentations de Gregor Mendel sur le développement des plantes hybrides (2e volet). L'Observatoire de la génétique.

[RSTA20090245c3] Auffray C., Noble D. (2009). Conceptual and experimental origins of integrative systems biology in William Harvey’s masterpiece on the movement of the heart and the blood in animals. Int. J. Mol. Sci..

[RSTA20090245c4] Auffray C., Nottale L. (2008). Scale relativity theory and integrative systems biology. I. Founding principles and scale laws. Prog. Biophys. Mol. Biol..

[RSTA20090245c5] Auffray C., Imbeaud S., Roux-Rouquie M., Hood L. (2003). Self-organized living systems:conjunction of a stable organization with chaotic fluctuations in biological space-time. Phil. Trans. R. Soc. Lond. A.

[RSTA20090245c6] Bard J. B. L. (2008). Waddington’s legacy to developmental and theoretical biology. Biol. Theory.

[RSTA20090245c7] Bassingthwaighte J. B., Hunter P. J., Noble D. (2009). The cardiac physiome:perspectives for the future. Exp. Physiol..

[RSTA20090245c8] Bernard C. (1865). Introduction à l’étude de la médecine expérimentale.

[RSTA20090245c9] Bird A. (2007). Perceptions of epigenetics. Nature.

[RSTA20090245c10] Bray D. (2009). Wetware. A computer in every cell.

[RSTA20090245c11] Bray D., Lay S. (1994). Computer simulated evolution of a network of cell-signalling molecules. Biophys. J..

[RSTA20090245c12] Cavalier-Smith T. (2000). Membrane heredity and early chloroplast evolution. Trends Plant Sci..

[RSTA20090245c13] Cavalier-Smith T., Hirt R. P., Horner D. S. (2004). The membranome and membrane heredity in development and evolution. Organelles, genomes and eukaryote phylogeny:an evolutionary synthesis in the age of genomics.

[RSTA20090245c14] Chen T., Zhang Y.-L., Jiang Y., Liu J.-H., Schatten H., Chen D.-Y., Sun Q.-Y. (2006). Interspecies nuclear transfer reveals that demethylation of specific repetitive sequences is determined by recipient ooplasm but not by donor intrinsic property in cloned embryos. Mol. Reprod. Dev..

[RSTA20090245c15] Chung Y. (2009). Reprogramming of human somatic cells using human and animal oocytes. Cloning Stem Cells.

[RSTA20090245c16] Clancy C. E., Rudy Y. (1999). Linking a genetic defect to its cellular phenotype in a cardiac arrhythmia. Nature.

[RSTA20090245c17] Clapworthy G., Viceconti M., Coveney P., Kohl P. (2008). Editorial. Phil. Trans. R. Soc. A.

[RSTA20090245c18] Coen E. (1999). The art of genes.

[RSTA20090245c19] Corsi P. (2001). Lamarck. Genèse et enjeux du transformisme.

[RSTA20090245c20] Crick F. H. C. (1958). On protein synthesis. Symp. Soc. Exp. Biol..

[RSTA20090245c21] Crick F. H. C. (1970). Central dogma of molecular biology. Nature.

[RSTA20090245c22] Davidson E. H. (2006). The regulatory genome:gene regulatory networks in development and evolution..

[RSTA20090245c23] Dawkins R. (1976). The selfish gene.

[RSTA20090245c24] Dawkins R. (1982). The extended phenotype.

[RSTA20090245c25] Dawkins R. (2006). The selfish gene.

[RSTA20090245c26] Dronamrajua K. R. (1999). Erwin Schrödinger and the origins of molecular biology. Genetics.

[RSTA20090245c27] Dupré J. (1993). The disorder of things.

[RSTA20090245c28] Fenner J. W. (2008). The EuroPhysiome, STEP and a roadmap for the virtual physiological human. Phil. Trans. R. Soc. A.

[RSTA20090245c29] Franklin R. E., Gosling R. G. (1953a). Evidence for 2-chain helix in crystalline structure of sodium deoxyribonucleate. Nature.

[RSTA20090245c30] Franklin R. E., Gosling R. G. (1953b). Molecular configuration in sodium thymonucleate. Nature.

[RSTA20090245c31] Garny A., Noble D., Kohl P. (2005). Dimensionality in cardiac modelling. Prog. Biophys. Mol. Biol..

[RSTA20090245c32] Goldenfeld N., Woese C. (2007). Biology’s next revolution. Nature.

[RSTA20090245c33] Hillenmeyer M. E. (2008). The chemical genomic portrait of yeast:uncovering a phenotype for all genes. Science.

[RSTA20090245c34] Hodgkin A. L., Huxley A. F. (1952). A quantitative description of membrane current and its application to conduction and excitation in nerve. J. Physiol..

[RSTA20090245c35] Hunter P. J., Pullan A. J., Smaill B. H. (2003). Modelling total heart function. Rev. Biomed. Eng..

[RSTA20090245c36] Huxley J. S. (1942). Evolution:the modern synthesis.

[RSTA20090245c37] Jablonka E., Lamb M. (1995). Epigenetic inheritance and evolution. The Lamarckian dimension.

[RSTA20090245c38] Jablonka E., Lamb M. (2005). Evolution in four dimensions.

[RSTA20090245c39] Jacob F. (1982). The possible and the actual.

[RSTA20090245c40] Jacob F., Perrin D., Sanchez C., Monod J., Edelstein S. (1960). The operon:a group of genes with expression coordinated by an operator. C. R. Acad. Sci. Paris.

[RSTA20090245c41] Kaern M., Elston T. C., Blake W. J., Collins J. J. (2005). Stochasticity in gene expression:from theories to phenotypes. Nat. Rev. Genet..

[RSTA20090245c42] Keller E. F. (1983). A feeling for the organism:the life and work of Barbara McClintock..

[RSTA20090245c43] Keller E. F. (2000a). The century of the gene.

[RSTA20090245c44] Keller E. F., Sloan P. R. (2000b). Is there an organism in this text?. Controlling our destinies. historical, philosophical, ethical and theological perspectives on the human genome project.

[RSTA20090245c45] Keller L. (1999). Levels of selection in evolution.

[RSTA20090245c46] Kitcher P. (1982). Genes. Br. J. Phil. Sci..

[RSTA20090245c47] Kohl P., Noble D. (2009). Systems biology and the virtual physiological human. Mol. Syst. Biol..

[RSTA20090245c48] Kupiec J.-J. (2008). L’origine des individus.

[RSTA20090245c49] Kupiec J.-J. (2009). The origin of individuals:a Darwinian approach to developmental biology.

[RSTA20090245c50] Laforge B., Guez D., Martinez M., Kupiec J.-J. (2004). Modeling embryogenesis and cancer:an approach based on an equilibrium between the autostabilization of stochastic gene expression and the interdependence of cells for proliferation. Prog. Biophys. Mol. Biol..

[RSTA20090245c51] Lamarck J.-B. (1809). Philosophie Zoologique.

[RSTA20090245c52] Levine M., Davidson E. H. (2005). Gene regulatory networks for development. Proc. Natl Acad. Sci. USA.

[RSTA20090245c53] Margulis L. (1981). Symbiosis in cell evolution.

[RSTA20090245c54] Margulis L., Sagan D. (2002). Acquiring genomes.

[RSTA20090245c55] Maynard Smith J., Szathmáry E. (1995). The major transitions in evolution.

[RSTA20090245c56] Mayr E. (1982). The growth of biological thought.

[RSTA20090245c57] Neuman Y. (2008). Reviving the living:meaning making in living systems.

[RSTA20090245c58] Noble D. (1960). Cardiac action and pacemaker potentials based on the Hodgkin-Huxley equations. Nature.

[RSTA20090245c59] Noble D. (1962). A modification of the Hodgkin-Huxley equations applicable to Purkinje fibre action and pacemaker potentials. J. Physiol..

[RSTA20090245c60] Noble D. (2006). The music of life.

[RSTA20090245c61] Noble D. (2008a). Claude Bernard, the first systems biologist, and the future of physiology. Exp. Physiol..

[RSTA20090245c62] Noble D. (2008b). Computational models of the heart and their use in assessing the actions of drugs. J. Pharmacol. Sci..

[RSTA20090245c63] Noble D. (2008c). Genes and causation. Phil. Trans. R. Soc. A.

[RSTA20090245c64] Nottale L. (1993). Fractal space-time and microphysics:towards a theory of scale relativity.

[RSTA20090245c65] Nottale L. (2000). La relativité dans tous ses états. Du mouvements aux changements d’échelle.

[RSTA20090245c66] Nottale L., Auffray C. (2008). Scale relativity and integrative systems biology. II. Macroscopic quantum-type mechanics. Prog. Biophys. Mol. Biol..

[RSTA20090245c67] Pearson H. (2006). What is a gene. Nature.

[RSTA20090245c68] Pichot A. (1999). Histoire de la notion de gène.

[RSTA20090245c69] Plank G. (2009). Generation of histo-anatomically representative models of the individual heart:tools and application. Phil. Trans. R. Soc. A.

[RSTA20090245c70] Saks V., Monge C., Guzun R. (2009). Philosophical basis and some historical aspects of systems biology:from Hegel to Noble—applications for bioenergetic research. Int. J. Mol. Sci..

[RSTA20090245c71] Scherrer K., Jost J. (2007). Gene and genome concept. Coding versus regulation. Theory Biosci..

[RSTA20090245c72] Schrödinger E. (1944). What is life?.

[RSTA20090245c73] Shapiro J. A. (2005). A 21st century view of evolution:genome system architecture, repetitive DNA, and natural genetic engineering. Gene.

[RSTA20090245c74] Shapiro J. A. (2009). Revisiting the central dogma in the 21st century. Ann. N Y Acad. Sci..

[RSTA20090245c75] Siegelmann H. T. (1995). Computation beyond the Turing Limit. Science.

[RSTA20090245c76] Siegelmann H. T. (1998). Neural networks and analog computation:beyond the Turing limit.

[RSTA20090245c77] Siegelmann H. T. (1999). Stochastic analog networks and computational complexity. J. Complexity.

[RSTA20090245c78] Strathmann R. R. (1993). Larvae and evolution:towards a new zoology. Q. Rev. Biol..

[RSTA20090245c79] Sulston J., Ferry G. (2002). The common thread.

[RSTA20090245c80] Sun Y. H., Chen S. P., Wang Y. P., Hu W., Zhu Z. Y. (2005). Cytoplasmic impact on cross-genus cloned fish derived from transgenic common carp (Cyprinus carpio) nuclei and goldfish (Carassius auratus) enucleated eggs. Biol. Reprod..

[RSTA20090245c81] Venter C. (2007). A life decoded.

[RSTA20090245c82] Waddington C. H. (1942). Canalization of development and the inheritance of acquired characteristics. Nature.

[RSTA20090245c83] Waddington C. H. (1957). The strategy of the genes.

[RSTA20090245c84] Waddington C. H. (1959). Canalization of development and genetic assimilation of acquired characteristics. Nature.

[RSTA20090245c85] Watson J. D., Crick F. H. C. (1953a). Genetical implications of the structure of deoxyribonucleic acid. Nature.

[RSTA20090245c86] Watson J. D., Crick F. H. C. (1953b). Molecular structure of nucleic acids. A structure for deoxyribose nucleic acid. Nature.

[RSTA20090245c87] Weaver I. C. G., Janigro D. (2009). Life at the interface between a dynamic environment and a fixed genome. Mammalian brain development.

[RSTA20090245c88] Weaver I. C. G., Cervoni N., Champagne F. A., D’Alessio A. C., Sharma S., Sekl J. R., Dymov S., Szyf M., Meaney M. J. (2004). Epigenetic programming by maternal behavior. Nat. Neurosci..

[RSTA20090245c89] Weaver I. C. G., D’Alessio A. C., Brown S. E., Hellstrom I. C., Dymov S., Sharma S., Szyf M., Meaney M. J. (2007). The transcription factor nerve growth factor-inducible protein a mediates epigenetic programming:altering epigenetic marks by immediate-early genes. J. Neurosci..

[RSTA20090245c90] Wilkins M. H. F., Stokes A. R., Wilson H. R. (1953). Molecular structure of deoxypentose nucleic acids. Nature.

[RSTA20090245c91] Williamson D. I. (2003). The origins of larvae.

[RSTA20090245c92] Williamson D. I. (2006). Hybridization in the evolution of animal form and life cycle. Zool. J. Linn. Soc..

[RSTA20090245c93] Williamson D. I., Vickers S. E. (2007). The origins of larvae. Am. Sci..

